# Effect of esketamine on postoperative depressive symptoms in patients undergoing thoracoscopic lung cancer surgery: A randomized controlled trial

**DOI:** 10.3389/fpsyt.2023.1128406

**Published:** 2023-03-15

**Authors:** Shu-lin Gan, Yu-qin Long, Qin-yun Wang, Chang-dong Feng, Chen-xu Lai, Chun-tong Liu, Yun-ying Ding, Hong Liu, Ke Peng, Fu-hai Ji

**Affiliations:** ^1^Department of Anesthesiology, First Affiliated Hospital of Soochow University, Suzhou, Jiangsu, China; ^2^Institute of Anesthesiology, Soochow University, Suzhou, Jiangsu, China; ^3^Department of Anesthesiology and Pain Medicine, University of California Davis Health, Sacramento, CA, United States

**Keywords:** anxiety, depression, esketamine, lung cancer, thoracoscopic surgery

## Abstract

**Background:**

Depressive symptoms are common among patients with lung cancer. We aimed to assess the effects of esketamine on postoperative depressive symptoms after thoracoscopic lung cancer surgery.

**Methods:**

In this randomized, double-blind, placebo-controlled trial, 156 patients undergoing thoracoscopic lung cancer surgery were randomly allocated in a 1:1 ratio to receive intravenous esketamine (intraoperatively and in patient-controlled analgesia until 48 h postoperatively) or normal saline placebo. The primary outcome was the proportion of patients with depressive symptoms at 1 month postoperatively, assessed using the Beck Depression Inventory-II (BDI-II). Secondary outcomes included depressive symptoms at 48 h postoperatively, hospital discharge and 3 months postoperatively, BDI-II scores, anxious symptoms, Beck Anxiety Inventory scores, Quality of Recovery-15 (QoR-15) scores, and 1- and 3-month mortality.

**Main results:**

A total of 151 patients (75 in the esketamine group and 76 in the normal saline group) completed the 1-month follow-up. The esketamine group had a significantly lower incidence of depressive symptoms at 1 month compared to the normal saline group (1.3% vs. 11.8%; risk difference = −10.5, 95%CI = −19.6% to −0.49%; *p* = 0.018). After excluding patients without lung cancer diagnosis, the incidence of depressive symptoms was also lower in the esketamine group (1.4% vs. 12.2%; risk difference = −10.8, 95%CI = −20.2% to −0.52%; *p* = 0.018). The secondary outcomes were similar between groups, except that the esketamine group had higher QoR-15 scores at 1 month postoperatively (median difference = 2; 95%CI = 0 to 5; *p* = 0.048). The independent risk factors for depressive symptoms were hypertension (odds ratio = 6.75, 95%CI = 1.13 to 40.31; *p* = 0.036) and preoperative anxious symptoms (odds ratio = 23.83, 95%CI = 3.41 to 166.33; *p* = 0.001).

**Conclusion:**

Perioperative administration of esketamine reduced the incidence of depressive symptoms at 1 month after thoracoscopic lung cancer surgery. History of hypertension and preoperative anxious symptoms were independent risk factors for depressive symptoms.

**Clinical trial registration:** Chinese Clinical Trial Registry http://www.chictr.org.cn, Identifier (ChiCTR2100046194).

## Introduction

According to the Global Cancer Statistics 2020, lung cancer is the second most common cancer and remains the leading cause of cancer death (1). Patients with lung cancer often suffer from psychiatric problems including depression and anxiety. A study showed that the incidence of anxiety and depression in lung cancer patients was 8 and 12% before surgery, respectively, which changed to 9 and 19% after surgery (2). Another study reported that the prevalence of depression was 8% at 1 month, 5.2% at 3 months, and 4.7% at 1 year after curative resection of non–small-cell lung cancer (3). These symptoms can be caused by chemicals released from lung cancer and treatments such as chemotherapy and corticosteroids (4, 5). Depression and anxiety are associated with poor adherence to treatments, reduced quality of life, prolonged hospital stay, and increased postoperative mortality (6). However, these psychiatric illnesses are often neglected, and there is no report on perioperative interventions to reduce depression and anxiety in patients undergoing thoracoscopic lung cancer surgery.

Ketamine is a racemic mixture of S-ketamine and R-ketamine enantiomers (7). Esketamine is the S-ketamine enantiomer, with more potent analgesic and anesthetic effects. Studies have reported the antidepressant effects of ketamine and esketamine. In patients with treatment-resistant depression, 72% of them responded to the ketamine treatment (8). A multicenter study found that the subanesthetic doses of ketamine vs. normal saline did not prevent depressive symptoms at 3 days (14.7% vs. 17.8%) in elderly patients after major surgery (9). In that study, the proportion of patients with depressive symptoms decreased from 17.5 to 6.9% at 30 days postoperatively, without significant difference after sensitivity analysis. A recent meta-analysis showed that intravenous ketamine was associated with reduced postoperative depressive symptoms within 3 days after surgery (10). Esketamine has been shown to reduce postoperative depression scores from 3 days to 1 month postoperatively in patients with breast cancer (11), while a recent study found that continuous intraoperative esketamine infusion did not reduce depression scores at 3 days after gynecological laparoscopy (12). Therefore, further investigations are still required to ascertain the effects of ketamine or esketamine on depressive symptoms in surgical patients.

The use of ketamine is associated with side effects such as nausea and vomiting, headache, and hallucinations (9, 10). Preclinical studies suggested that those side effects of ketamine may be attributed to S-ketamine, but not R-ketamine (7, 13). However, recent clinical evidence showed that perioperative use of ketamine increased the risk of psychotomimetic adverse events (14), while esketamine did not significantly increase the risk (12, 15).

For the assessment of depressive symptoms, Montgomery-Asberg Depression Rating Scale, 9-item Patient Health Questionnaire, Hamilton Depression Scale and Beck Depression Inventory (BDI) have been used in clinical settings (16–18). Of them, BDI is a validated and commonly used measure of depressive symptoms. In this study, we investigated the effects of esketamine on postoperative psychiatric symptoms and related side effects in patients undergoing lung cancer surgery. We hypothesized that perioperative administration of esketamine would improve postoperative depressive symptoms, anxious symptoms, and recovery after thoracoscopic lung cancer surgery.

## Methods

### Study design and ethics

This single-center, randomized, double-blind, placebo-controlled trial was conducted at the First Affiliated Hospital of Soochow University, Suzhou, Jiangsu, China. The study protocol was approved by the Ethics Committee of the First Affiliated Hospital of Soochow University (Approval No. 2020-127). This study was registered on the Chinese Clinical Trial Registry on May 9, 2021 before the enrollment of the first participant. All patients provided their written informed consent. This study was performed following the Declaration of Helsinki and the Consolidated Standards of Reporting Trials guidelines.

### Patients

Patients aged ≥18 years with American Society of Anesthesiologists (ASA) physical status I–III scheduled for thoracoscopic lung cancer surgery were eligible for inclusion. Preoperative diagnosis of lung cancer was based on computed tomography or pathological results. The exclusion criteria included:

1. non-tumor diseases, such as lung infection, fibrosis, and tuberculosis;
2. preoperative mental illness or use of antipsychotics;
3. serious cardiac, renal and liver diseases (heart failure, myocardial infarction, left ventricular ejection fraction <30%, Child–Pugh grade C, and renal replacement therapy);
4. inability to communicate, read, or write;
5. allergies to study medications; or
6. refusal to participate in this study.

### Randomization and blinding

Based on a central randomization system,^1^ patients were randomly allocated in a 1:1 ratio to either the esketamine group or the normal saline group. The details of allocation were concealed using password protection. A research nurse not involved in the subsequent study prepared the study medications according to the randomization results. Both esketamine and normal saline placebo are clear and colorless fluids, so there is no way to distinguish them. All patients, anesthesiologists, surgeons, perioperative care providers, and postoperative assessors were blinded to the group allocation until the final statistical analysis.

### Anesthetic care

After entering the operating room, patients were monitored with electrocardiography, non-invasive blood pressure, and peripheral oxygen saturation (SpO_2_). Under local anesthesia with 2% lidocaine, an arterial line was placed *via* the radial artery for continuous arterial pressure monitoring and blood sampling. Anesthesia depth was assessed using the bispectral index (BIS). Anesthesia was induced with intravenous propofol 1.5–2 mg/kg, sufentanil 0.3–0.5 μg/kg, and cisatracurium 0.2 mg/kg. Patients were intubated with a double-lumen endotracheal tube, and the location was confirmed by capnography, auscultation and fiberoptic bronchoscopy. In the lateral position, single-lung ventilation was started (tidal volume of 4–6 ml/kg, respiratory rate of 12–20 breaths/min, 60–100% oxygen, and positive end-expiratory pressure of 5–10 cm H_2_O) to maintain end-tidal carbon dioxide within 35–45 mmHg and SpO_2_ ≥ 90%. Lung recruitment maneuvers were carried out when indicated. Anesthesia was maintained with sevoflurane 1–3% inhalation, titrated to BIS values within 40–60. Intermittent boluses of sufentanil or cisatracurium were given intravenously when needed. Patients received Lactated Ringer’s solution for intraoperative volume repletion. Nasopharyngeal temperature was maintained within 36–37°C using a warming blanket.

Hypotension was defined as mean arterial pressure (MAP) < 65 mmHg and treated with fluid infusion, ephedrine, or phenylephrine. Bradycardia was defined as heart rate (HR) < 50 beats/min and treated with intravenous atropine. Interventions for hypertension (defined as MAP >90 mmHg) and tachycardia (defined as HR > 100 beats/min) included adjustment of anesthesia and administration of medications (nitroglycerine, nicardipine or esmolol). At the end of surgery, all patients received intravenous flurbiprofen axetil 50 mg, ondansetron 4 mg, and wound infiltration with 0.5% ropivacaine 15–20 ml. Patients were transferred to the post-anesthetic care unit and extubated there.

### Study interventions

In the esketamine group, esketamine 0.1 mg/kg was administered intravenously at about 3 min before skin incision, followed by a continuous infusion of 0.1 mg/kg/h until the end of surgery. The normal saline group received the same volume of normal saline placebo. Patients received sufentanil-based patient-controlled intravenous analgesia (PCIA) during the first 48 h postoperatively. In the PCIA device, sufentanil 2 μg/kg combined with esketamine 1 mg/kg (the esketamine group) or sufentanil 2 μg/kg only (the normal saline group) was diluted with normal saline to a final volume of 100 ml. The background infusion rate was 1.5 ml/h, the bolus dose was 1.5 ml, and the lockout interval was 10 min. Thus, we used esketamine 0.1 mg/kg bolus +0.1 mg/kg/h intraoperatively, followed by 0.015 mg/kg/h postoperatively for 48 h. This dosing regimen of esketamine was according to our clinical practice and the recent literature in which esketamine was administered as 0.125 to 0.25 mg/kg bolus, with or without 0.015 to 0.125 mg/kg/h for 48 h (11, 19, 20).

### Study outcomes

The primary outcome was the incidence of depressive symptoms at 1 month postoperatively. An independent investigator assessed the depressive symptoms using a WeChat-based questionnaire of the Beck Depression Inventory-II (BDI-II) (18). The BDI-II is a validated self-report measure of depressive symptoms, consisting of 21 items (3 points for each item and scores ranging from 0–63) (21). A total score of 0–13, 14–19, 20–28, and 29–63 indicates absent, mild, moderate and severe depressive symptoms, respectively (22). Patients completed the questionnaire in their own time to avoid the possibility of observer bias (23).

The secondary outcomes included BDI-II scores and depressive symptoms incidence at 48 h postoperatively, at hospital discharge, and at 3 months postoperatively, BDI-II scores at 1 month postoperatively, Beck Anxiety Inventory (BAI; 21 items with scores ranging from 0–63) (24) scores and anxious symptoms incidence at 48 h postoperatively and at hospital discharge, Quality of Recovery-15 (QoR-15) (25) scores at 48 h postoperatively, at hospital discharge, and at 1 month postoperatively, and postoperative 1- and 3-month mortality. BAI and QoR-15 questionnaires were also completed *via* the WeChat application. Studies have validated the Chinese versions of BDI, BAI, and QoR-15 (26–28).

### Data collection

Patients’ demographics and baseline characteristics were collected 1 day before surgery. Patients were assessed for preoperative depressive and anxious symptoms using BDI-II and BAI. Intraoperative data included use of anesthetics and analgesics (propofol, sevoflurane, sufentanil and esketamine), hemodynamic events, blood loss, fluid infusion, urine output, type of surgery, duration of surgery, and duration of anesthesia. Postoperative data included pain scores at rest and on coughing at 24 and 48 h postoperatively, patient-controlled sufentanil consumption within 0–24 h and 0–48 h postoperatively, esketamine consumption during 0–48 h postoperatively, adverse events (postoperative nausea and vomiting [PONV], nightmare, dizziness, blurred vision, dissociation and delirium), and length of postoperative hospital stay. Pain intensity was assessed using the visual analog scale (VAS; ranging from 0–10, higher scores indicate more pain). Delirium was assessed using the Confusion Assessment Method (CAM) or Confusion Assessment Method for Intensive Care Unit (CAM-ICU) (29). Patients were followed up at 1 month and 3 months postoperatively *via* WeChat questionnaires.

### Sample size estimation

A recent study reported that the incidence of postoperative depressive symptoms was 19% in patients undergoing surgical treatment for lung cancer (30). The effect of esketamine on depressive symptoms after lung cancer surgery is unknown. A recent study showed that a low-dose ketamine compared to normal saline placebo reduced the occurrence of depressive symptoms by 61% (from 17.5 to 6.9%) at 30 days after major surgery in older adults (9). In addition, another study suggested that the postoperative depression scores were lower in the esketamine group than those in the ketamine group for patients with breast cancer (11). Based on these, we hypothesized that use of esketamine could reduce the depressive symptoms by 70% (i.e., from 19 to 5.7%) in our patients. Thus, 70 patients in each group were required with one-sided test at α level of 0.05 and power of 80%. To allow for a 10% dropout rate, we finally enrolled 156 patients with 78 in each group. We calculated the required sample size using the PASS software (NCSS, LCC, Kaysville, UT).

### Statistical analysis

Data are expressed as mean [standard deviation (SD)], median [inter-quartile range (IQR)], or numbers (percentages). Continuous data were checked for normality using the Kolmogorov–Smirnov test and analyzed using the unpaired Student t-test or Mann–Whitney rank–sum test. Categorical data were analyzed with the chi-square test or Fisher exact test. For the primary and secondary outcomes, the between-group differences were analyzed using risk difference or median difference with 95% confidence interval (CI).

The primary analyzes were conducted in the modified intention-to-treat population including patients who underwent randomization and surgery with available primary outcome measurement (1-month depressive symptoms rate). As depressive symptoms were associated with the pathological results, the primary outcome was also analyzed after excluding patients without lung cancer diagnosis.

We analyzed the risk factors for depressive symptoms at 1 month postoperatively in the modified intention-to-treat population. First, we applied the univariate logistic regression analysis for possible predictive factors. We chose the variables with *p* < 0.25 based on statistical significance and clinical relevance, and we included these variables into the multiple regression analysis. We analyzed the variance inflation factor to assess multicollinearity of the variables in the multiple regression model. The goodness of fit of the model was examined using the Hosmer–Lemeshow test.

No multiple testing corrections were planned for the secondary outcomes, so these results should be regarded exploratory. We did not perform an interim analysis. Missing values were not imputed. In addition, we conducted *post hoc* subgroup analysis for the 1-month depressive symptoms rate between male and female patients. Except for the subgroup analysis, all analyzes in this study were planned *a priori*. Statistical analyzes were performed with SPSS software (version 22.0, IBM SPSS, Chicago, IL). A two-sided *p* < 0.05 was considered statistically significant.

## Results

### Study flow

**Figure 1:**
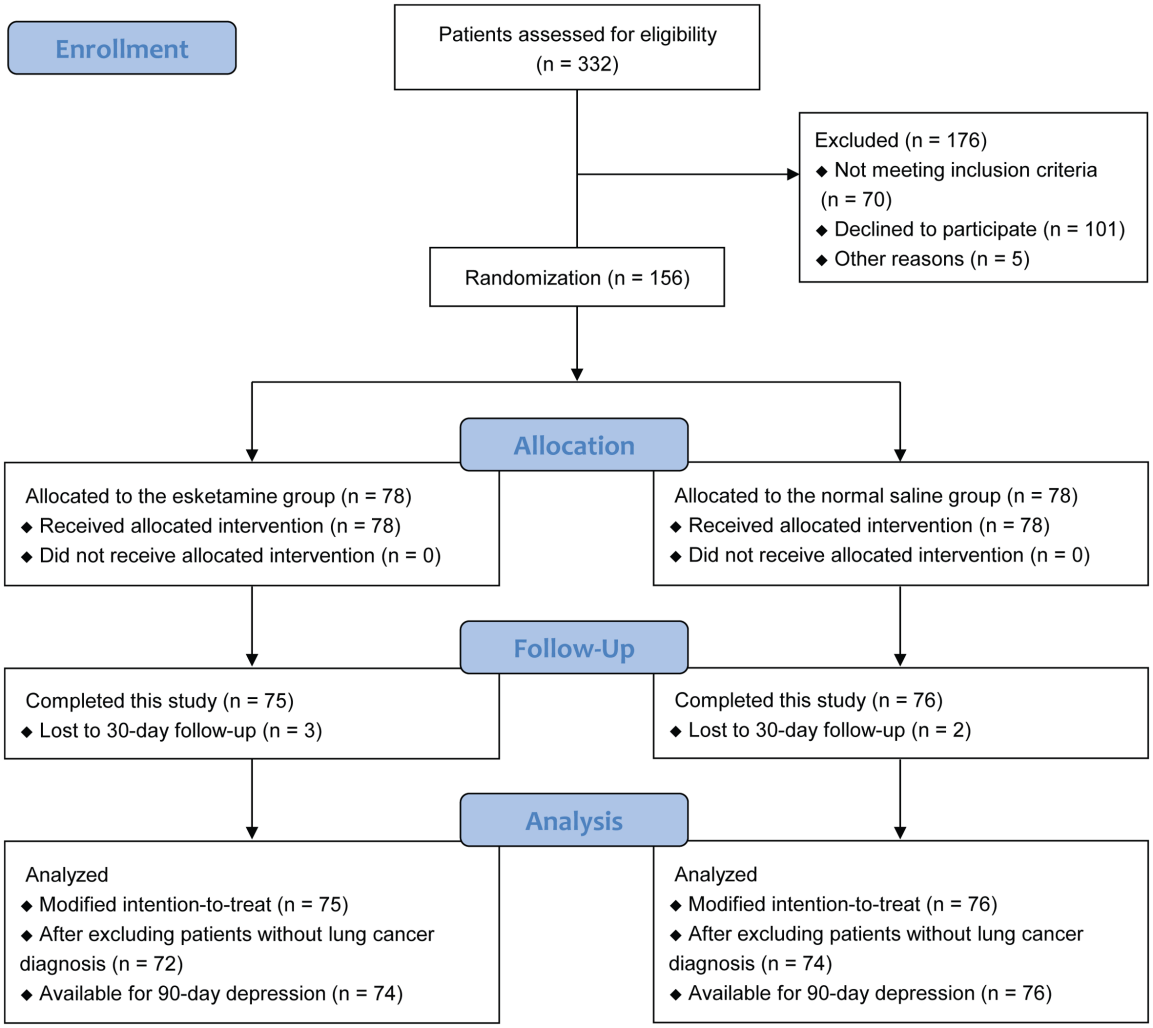
Flowchart of participants.

From May 18, 2021 to May 30, 2022, a total of 332 patients scheduled for thoracoscopic lung cancer surgery were screened (Figure 1). Of them, 176 patients were excluded due to not meeting the inclusion criteria (*n* = 70), refusal for participation (*n* = 101), and cancelation of surgery (*n* = 5). Thus, 156 patients were randomized, with 78 in each group. Five patients were lost to 30-day follow-up (3 in the esketamine group and 2 in the normal saline group). The modified intention-to-treat analyzes included 151 patients (75 in the esketamine group and 76 in the normal saline group) who had available primary outcome data. Analyzes were also performed after excluding patients without lung cancer diagnosis (3 in the esketamine group and 2 in the normal saline group). At 90 days postoperatively, 74 patients in the esketamine group and 76 in the normal saline group were assessed for depressive symptoms.

### Baseline data

The demographic and baseline characteristics were comparable between the two groups (Table 1). The mean (SD) age was 54 (12) years for both groups. 69.2% of patients in the esketamine group and 76.9% in the normal saline group were female sex. At 1 day before surgery, patients were assessed for depressive and anxious symptoms. Two patients in the normal saline group showed preoperative depressive symptoms; however, they had no history of previous depressive episodes, nor they were on antidepressants. Three patients in the esketamine group and 5 patients in the normal saline group exhibited anxious symptoms. The median (IQR) time between suspected diagnosis and surgery was 2.5 (0.6, 7.3) and 2 (0.5, 9.3) months in the esketamine group and the normal saline group, respectively.

**Table 1:** Demographic and baseline data.

	Esketamine group (*n* = 78)	Normal saline group (*n* = 78)	*p* value
Age, yr	54.0 (11.7)	53.8 (12.1)	0.904
Female sex	54 (69.2%)	60 (76.9%)	0.279
Body mass index, kg/m^2^	23.1 (2.7)	23.1 (2.6)	0.934
Education, yr	9 (3, 12)	9 (5, 12)	0.353
Smoker			0.823
Current	6 (7.7%)	5 (6.4%)	
Never	70 (89.7%)	72 (92.3%)	
Quit	2 (2.6%)	1 (1.3%)	
Marital status			1.000
Married or living as married	77 (98.7%)	76 (97.4%)	
Single	1 (1.3%)	2 (2.6%)	
Separated, divorced, or widowed	0	0	
Employment status			1.000
In paid employment	38 (48.7%)	38 (48.7%)	
Unemployment	40 (51.3%)	40 (51.3%)	
ASA			0.872
I	35 (44.9%)	32 (41%)	
II	42 (53.8%)	45 (57.7%)	
III	1 (1.3%)	1 (1.3%)	
Comorbidities			
Hypertension	23 (29.5%)	25 (32.1%)	0.729
Diabetes	9 (11.5%)	8 (10.3%)	0.797
Coronary heart disease	1 (1.3%)	1 (1.3%)	1.000
Stroke	1 (1.3%)	2 (2.6%)	1.000
COPD	0	0	1.000
Asthma	3 (3.8%)	1 (1.3%)	0.612
Thyroid disease	4 (5.1%)	5 (6.4%)	1.000
Previous cancer	4 (5.1%)	7 (9%)	0.348
Preoperative medication			
ACEIs/ARBs	9 (11.5%)	9 (11.5%)	1.000
Calcium channel blockers	7 (9%)	8 (10.3%)	0.786
Diuretics	5 (6.4%)	3 (3.8%)	0.717
Beta-blockers	4 (5.1%)	1 (1.3%)	0.363
Aspirin or antiplatelets	2 (2.6%)	2 (2.6%)	1.000
Depressive or anxious symptoms at 1 day before surgery			
Depressive symptoms	0	2 (2.6%)	0.477
Anxious symptoms	3 (3.8%)	5 (6.4%)	0.717
Tumor diameter, mm	11 (8, 20)	10 (8, 17.3)	0.784
Time between suspected diagnosis and surgery, mon	2.5 (0.6, 7.3)	2 (0.5, 9.3)	0.928

Data are mean (standard deviation), median (inter-quartile range), or *n* (%). ASA, American society of Anesthesiologists; COPD, chronic obstructive pulmonary disease; ACEI, angiotensin-converting enzyme inhibitors; ARB, angiotensin receptor blockers.

### Perioperative data

Table 2 shows the intraoperative and postoperative data. The two groups had similar intraoperative propofol, sevoflurane, and sufentanil consumption. The mean (SD) total dose of intraoperative esketamine was 21.6 (7.0) mg. The two groups were similar in terms of hemodynamic events, blood loss, fluid infusion, urine output, type of surgery, duration of surgery, or duration of anesthesia. For the postoperative data, the two groups had similar VAS pain scores and required similar amount of patient-controlled sufentanil. The mean (SD) total dose of postoperative esketamine was 45.1 (8.8) mg. Adverse events were comparable between groups. 43.6% of patients in both groups experienced PONV; most episodes were mild, and 3 patients required additional antiemetics. No patients experienced dissociation or delirium. The median (IQR) length of postoperative hospital stay was 4 (3.0, 6.3) days in the esketamine group and 5 (3, 6) days in the normal saline group.

**Table 2:** Intra- and postoperative data.

	Esketamine group (*n* = 78)	Normal saline group (*n* = 78)	*p* value
Intraoperative data			
Anesthetics and analgesics			
Propofol (mg)	130 (110, 150)	120 (100, 140)	0.056
Sevoflurane (MAC·h)	1.8 (0.7)	2.0 (0.8)	0.111
Intraoperative sufentanil (μg)	49.7 (8.2)	49.2 (8.2)	0.698
Esketamine (mg)	21.6 (7.0)	-	-
Hemodynamic events			
Hypotension	19 (24.4%)	15 (19.2%)	0.438
Hypertension	15 (19.2%)	7 (9.0%)	0.066
Bradycardia	8 (10.3%)	6 (7.7%)	0.575
Tachycardia	3 (3.8%)	2 (2.6%)	1.000
Blood loss (ml)	40 (20, 50)	30 (20, 50)	0.794
Fluid infusion (ml)	900 (700, 1,000)	900 (700, 1,300)	0.439
Urine output (ml)	250 (138, 400.0)	300 (200, 400)	0.191
Type of surgery			0.193
Wedge resection	30 (38.5%)	21 (26.9%)	
Segmentectomy	15 (19.2%)	23 (29.5%)	
Lobectomy	33 (42.3%)	34 (43.6%)	
Duration of surgery (min)	125 (79.3, 165)	132 (91.8, 195.5)	0.202
Duration of anesthesia (min)	145 (105, 196.3)	164 (118.8, 220.5)	0.160
Postoperative data			
VAS pain scores			
At rest at 24 h postoperatively	0 (0, 1)	0 (0, 1)	0.807
On coughing at 24 h postoperatively	3 (0, 5)	3 (2, 5)	0.556
At rest at 48 h postoperatively	0 (0, 1)	0 (0, 0)	0.692
On coughing at 48 h postoperatively	3 (3, 6)	3 (2, 4)	0.140
Sufentanil 0–24 h after surgery (μg)	42.2 (37.8, 52.5)	42 (38.5, 49.3)	0.887
Sufentanil 0–48 h after surgery (μg)	87.8 (77.1, 101.6)	85.2 (78.1, 99.4)	0.477
Esketamine 0–48 h after surgery (mg)	45.1 (8.8)	**-**	**-**
Adverse events within 0–48 h postoperatively			
PONV	34 (43.6%)	34 (43.6%)	1.000
Nightmare	1 (1.3%)	4 (5.1%)	0.363
Dizziness	18 (23.1%)	17 (21.8%)	0.848
Blurred vision	1 (1.3%)	2 (2.6%)	1.000
Dissociation	0	0	1.000
Delirium	0	0	1.000
Postoperative hospital stay (d)	4 (3.0, 6.3)	5 (3, 6)	0.631

Data are mean (standard deviation), median (inter-quartile range), or *n* (%). MAC, minimum alveolar concentration; VAS, visual analog scale; PONV, postoperative nausea and vomiting.

### Primary outcome

**Figure 2:**
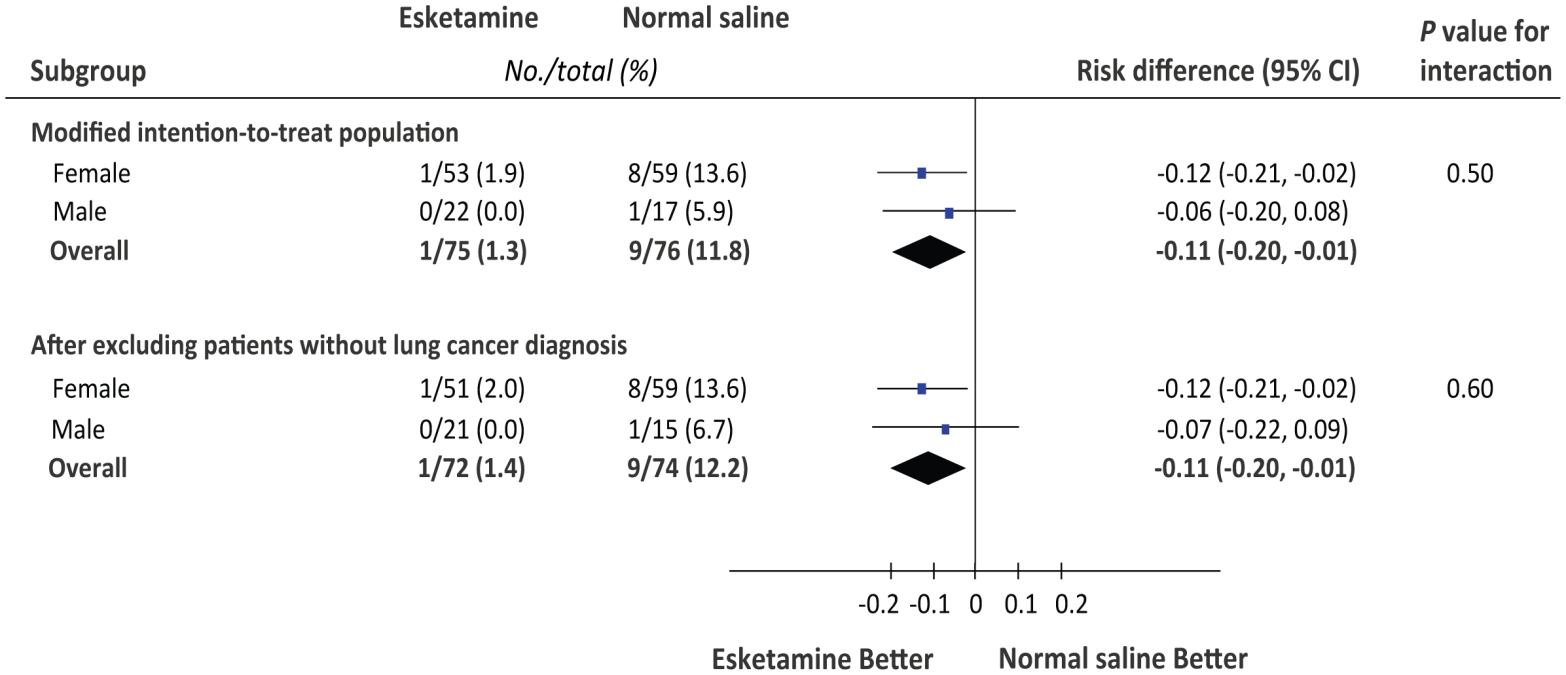
*Post hoc* subgroup analysis for the 1-month depression rate between males and females.

The primary outcome of depressive symptoms at 1 month postoperatively was assessed in two study populations, respectively, (Table 3). In the modified intention-to-treat population, patients receiving esketamine had a significantly lower incidence of depressive symptoms at 1 month postoperatively than those receiving normal saline placebo (1.3% vs. 11.8%; risk difference = −10.5, 95%CI = −19.6% to −0.49%; *p* = 0.018). Postoperative pathological diagnosis confirmed non-cancerous lesions but not lung cancer in 5 patients (3 in the esketamine group and 2 in the normal saline group). After excluding these patients, the incidence of depressive symptoms was also lower in the esketamine group (1.4% vs. 12.2%; risk difference = −10.8, 95%CI = −20.2% to −0.52%; *p* = 0.018). The *post hoc* subgroup analysis showed that the 1-month depressive symptoms rate did not differ significantly between males and females (Figure 2).

**Table 3:** Primary and secondary outcomes.

	Esketamine group (*n* = 78)	Normal saline group (*n* = 78)	Risk or median difference (95%CI)	*P* value
Primary outcome depressive symptoms at 1 month postoperatively				
Modified intention-to-treat population	1 (1.3%) (n = 75)	9 (11.8%) (n = 76)	−10.5% (−19.6% to −0.49%)	0.018
After excluding patients without lung cancer diagnosis	1 (1.4%) (n = 72)	9 (12.2%) (n = 74)	−10.8% (−20.2% to −0.52%)	0.018
Secondary outcomes				
Depressive symptoms				
BDI-II score at 48 h postoperatively	6 (5, 8)	6 (5, 8)	0 (−1 to 0)	0.433
Depressive symptoms at 48 h postoperatively	0	1 (1.3%)	−1.3% (−7.3 to 5.4%)	1.000
BDI-II score at hospital discharge	6 (5, 7)	6 (5, 8)	0 (−1 to 0)	0.396
Depressive symptoms at hospital discharge	0	4 (5.1%)	−5.1% (−11.9 to 3.1%)	0.120
BDI-II score at 1 month postoperatively	5 (4, 7) (n = 75)	5 (4, 8) (n = 76)	0 (−1 to 1)	0.644
BDI-II score at 3 months postoperatively	1.5 (0, 4) (n = 74)	2 (0, 5) (n = 76)	0 (−1 to 1)	0.180
Depressive symptoms at 3 months postoperatively	0 (n = 74)	1 (1.3%) (n = 76)	−1.3% (−7.6 to 5.5%)	1.000
Anxious symptoms				
BAI score at 48 h postoperatively	1 (1, 2)	1 (1, 2)	0 (0 to 0)	0.752
Anxious symptoms at 48 h postoperatively	0	2 (2.6%)	−2.6% (−8.8 to 4.7%)	0.497
BAI score at hospital discharge	1 (0, 2)	1 (0.8, 2.0)	0 (0 to 0)	0.736
Anxious symptoms at hospital discharge	0	2 (2.6%)	−2.6% (−8.8 to 4.7%)	0.497
Recovery				
QoR-15 score at 48 h postoperatively	119.5 (109.5, 128.3)	118 (105, 130)	1 (−4 to 6)	0.714
QoR-15 score at hospital discharge	124 (115.8, 131.3)	124.5 (111.8, 131.0)	1 (−4 to 5)	0.690
QoR-15 score at 1 month postoperatively	140 (136, 144) (n = 75)	139 (132, 142) (n = 76)	2 (0 to 5)	0.048
Mortality				
1-month	0 (n = 75)	0 (n = 76)	0% (−6.0 to 6.0%)	1.000
3-month	1 (1.3%) (n = 75)	0 (n = 76)	1.3% (−4.8 to 8.2%)	0.497

Data are median (inter-quartile range) or *n* (%). BDI-II, Beck Depression Inventory-II; BAI, Beck Anxiety Inventory; QoR-15, Quality of Recovery-15.

The details of patients with depressive symptoms are presented in Supplementary Table S1. These patients aged between 34 and 70 years, and most of them (9 out of 10) were female sex. At 1 month postoperatively, the depressive symptoms were mild in 7 patients, experienced moderate in 2 patients, and severe in 1 patient. Two patients with preoperative depressive symptoms (case 6 and case 9) still had symptoms at 1 month postoperatively. Three patients (cases 1, 3, and 6) needed postoperative psychological counseling and received antidepression medications.

### Secondary outcomes

The secondary outcomes are shown in Table 3. The two study groups were similar in terms of the BDI-II scores and incidence of depressive symptoms at 48 h postoperatively and at the time of hospital discharge. Of note, at 1 month postoperatively, the median (IQR) BDI-II score was 5 (4, 7) in the esketamine group, which was similar to 5 (4, 8) in the normal saline group (*p* = 0.664). At 3 months postoperatively, 1 patient in the normal saline group still had depressive symptoms. Two patients in the normal saline group showed anxious symptoms at 48 h postoperatively and at hospital discharge. Regarding the quality of recovery after surgery, the two groups had similar QoR-15 scores at 48 h postoperatively and at hospital discharge; however, the esketamine group had higher median QoR-15 scores at 1 month postoperatively compared to the normal saline group (median difference = 2, 95%CI = 0 to 5; *p* = 0.048). One patient in the esketamine group died at postoperative day 45 due to respiratory failure.

### Risk factors for depressive symptoms

Based on the univariate logistic regression analysis, female sex, in paid employment, history of hypertension, and preoperative anxious symptoms were associated with increased risk for depressive symptoms at 1 month postoperatively, while use of esketamine was associated with a lower depressive symptoms rate (Table 4). Thus, these 5 factors were included into the multiple regression model and analyzed as categorical variables.

**Table 4:** Univariate logistic regression analysis of possible predictive factors for depressive symptoms at 1 month postoperatively in the modified intention-to-treat population.

	Depressive symptoms (*n* = 10)	No depressive symptoms (*n* = 141)	Univariate odds ratio (95% CI)	*P* value
Age, yr				
≤50	5 (50%)	48 (34%)	Reference	
>50	5 (50%)	93 (66%)	0.52 (0.14 to 1.87)	0.314
Sex				
Male	1 (10%)	39 (27.7%)	Reference	
Female	9 (90%)	102 (72.3%)	3.44 (0.42 to 28.06)	**0.248**
Education				
Elementary	4 (40%)	54 (38.3%)	Reference	
Middle school	3 (30%)	30 (21.3%)	1.35 (0.28 to 6.44)	0.707
High school	2 (20%)	29 (20.6%)	0.93 (0.16 to 5.39)	0.936
University/above	1 (10%)	28 (19.9%)	0.48 (0.05 to 4.52)	0.523
Employment status				
Unemployment	3 (30%)	72 (51.1%)	Reference	
In paid employment	7 (70%)	69 (48.9%)	2.44 (0.61 to 9.80)	**0.210**
History of hypertension	5 (50%)	41 (29.1%)	2.44 (0.67 to 8.88)	**0.176**
Preoperative depressive symptoms	2 (20%)	0	-	0.999
Preoperative anxious symptoms	4 (40%)	4 (2.8%)	22.83 (4.57 to 114.12)	**0.000**
Intervention				
Normal saline	9 (90%)	67 (47.5%)	Reference	
Esketamine	1 (10%)	74 (52.5%)	0.10 (0.01 to 0.82)	**0.031**

Data are *n* (%). Bold value means *P* < 0.25, and these factors were analyzed in Table 5.

In this multiple logistic regression analysis, the values of variance inflation factor were < 2 for all factors (1.058 for female sex, 1.126 for in paid employment, 1.167 for history of hypertension, 1.017 for preoperative anxious symptoms, and 1.012 for use of esketamine), which indicates minimal collinearity among these predictors. The Hosmer–Lemeshow test showed that *p* = 0.698, suggesting a high goodness of fit of our model. The independent risk factors for depressive symptoms at 1 month postoperatively were hypertension (multivariate odds ratio = 6.75, 95%CI = 1.13 to 40.31; *p* = 0.036) and preoperative anxious symptoms (multivariate odds ratio = 23.83, 95%CI = 3.41 to 166.33; *p* = 0.001). The use of esketamine was a protective factor against 1-month depressive symptoms postoperatively (multivariate odds ratio = 0.09, 95%CI = 0.01 to 0.91; *p* = 0.042; Table 5).

**Table 5:** Multiple logistic regression analysis of potential risk factors for depressive symptoms at 1 month postoperatively in the modified intention-to-treat population.

	Multivariate odds ratio (95% CI)	*P* value
Female	7.73 (0.41 to 144.05)	0.171
In paid employment	4.55 (0.71 to 28.95)	0.109
Hypertension	6.75 (1.13 to 40.31)	0.036
Preoperative anxious symptoms	23.83 (3.41 to 166.33)	0.001
Esketamine	0.09 (0.01 to 0.91)	0.042

## Discussion

This study showed that the perioperative infusion of esketamine prevented the occurrence of depressive symptoms at 1 month after thoracoscopic lung cancer surgery. This finding was evident in the modified intention-to-treat patient population, as well as after excluding patients without lung cancer diagnosis. Moreover, the QoR-15 scores at 1 month were slightly higher in patients receiving esketamine, suggesting that the improvement in depressive symptoms contributed to better postoperative recovery. Our results did not show between-group differences in anxious symptoms, and the use of esketamine did not induce concerning adverse events in our patients. The regression analyzes suggested that history of hypertension and preoperative anxious symptoms were independent risk factors for depressive symptoms and that the use of esketamine was a protective factor.

For patients who underwent lung cancer surgery, the incidence of postoperative depressive symptoms at 2 weeks after discharge was reported to be 19% (30). In our study, 11.8% of patients in the normal saline group developed depressive symptoms, whereas the esketamine treatment reduced the depressive symptoms rate to 1.3%. For patients who had breast cancer and preoperative mild/moderate depressive symptoms, the use of esketamine led to lower postoperative depression scores when compared to saline or racemic ketamine (11). Another recent study showed that esketamine alleviated short-term depressive symptoms after laparoscopic modified radical hysterectomy (19). Furthermore, several studies also suggested that the use of esketamine prevented postpartum depressive symptoms after cesarean section (31, 32). A recent study showed that intraoperative esketamine infusion improved postoperative sleep disturbance, but not depression symptoms, after gynecological laparoscopy (12). However, there is lack of evidence regarding the effects of esketamine on depression after cancer surgery. Our study is the first to demonstrate the impact of esketamine on depressive symptoms in patients who underwent lung cancer surgery.

Studies have suggested that perioperative use of esketamine was associated with reduced pain intensity and opioid requirements after surgery (12, 15, 20). However, our study did not show such a relationship. The two groups had similar pain scores and sufentanil consumption. Both groups reported low VAS pain scores, which can be attributable to the multimodal analgesia strategy used in all our patients (flurbiprofen axetil, wound infiltration with ropivacaine, and sufentanil-based PCIA). In terms of adverse effects, recent studies found that esketamine did not significantly increase the risk for nausea, vomiting, and psychotomimetic adverse events (12, 15). On the other hand, a recent meta-analysis showed that perioperative use of ketamine reduced postoperative depression scores, but increased the risk of adverse effects including nausea and vomiting, headache, dizziness, and hallucination (14). In our study, the most commonly reported adverse events were mild PONV and dizziness, and the rates were similar between groups. Events of nightmare and blurred vision were uncommon, and no patients had dissociation or delirium.

Recent experimental studies have explored the mechanisms underlying the antidepressant role of ketamine or its two enantiomers. Yu and colleagues found that ketamine produced antidepressant effects which was mediated by Neurocan in the prelimbic cortex of adolescent rats (33). Another study showed that the antidepressant-dose ketamine reversed the depressive behaviors in mice by restoring lost spines and rescuing coordinated ensemble activity (34). Rawat et al. revealed that the activation of immature granule neurons in the dentate gyrus of hippocampus was necessary and sufficient for the antidepressant effect of ketamine (35). In lipopolysaccharide-induced depression mice models, esketamine conveyed antidepressant effects by inhibiting autophagy *via* the mTOR- brain-derived neurotrophic factor (BDNF) pathway (36). For mice subjected to abdominal surgery, esketamine alleviated postoperative depression-like behaviors by inhibiting inflammatory effects in the prefrontal cortex (37). Preclinical studies showed that R-ketamine has greater potency and longer-lasting antidepressant effects than esketamine in animal models of depression. BDNF is the key to the antidepressant-like effects of racemic ketamine and its two enantiomers (38). R-ketamine enhanced BDNF transcription by activation of ERK-NRBP1-CREB signaling in microglia in chronic social defeat stress susceptible mice (38). In addition, another study reported that TGF-β1 signaling pathway plays a role in the antidepressant effect of R-ketamine (39).

The strengths of our study included the randomized, double-blind, controlled design, 3 months of follow-up, and the use of WeChat-based questionnaires. Questionnaires were completed by our patients *via* WeChat, which avoided the observer bias. In addition, we utilized regression models to determine the risk factors for depressive symptoms at 1 month postoperatively in the modified intention-to-treat population, and our results suggested that preoperative hypertension and anxious symptoms were independent risk factors and esketamine treatment was a protective factor. Last, we conducted *post hoc* subgroup analysis to explore the effects of esketamine between male and female patients.

Our study also has some limitations. First, although the number of included patients was consistent with the sample size calculation, including more patients may give the trial more power to test between-group differences. Second, this study was conducted at a single center situated in eastern China. Whether the results could be generalizable to other institutions needs further investigation. Third, the usage of esketamine in this study was based on the recent literature and our clinical practice, but it may not be definitive. In addition, we did not collect the pharmacokinetic data in our patients. Last, the follow-up period was limited to 3 months. Further studies are needed to ascertain the generalizability of our results and longer-term effects of esketamine in thoracoscopic lung cancer surgery.

In conclusion, the perioperative administration of esketamine reduced the incidence of depressive symptoms at 1 month postoperatively for patients undergoing thoracoscopic lung cancer surgery, without incurring significant adverse events. History of hypertension and preoperative anxious symptoms were independent risk factors for depressive symptoms.

## Data availability statement

The raw data supporting the conclusions of this article will be made available by the authors, without undue reservation.

## Ethics statement

This study involving human participants was reviewed and approved by the Ethics Committee of the First Affiliated Hospital of Soochow University (Approval No. 2020-127). All patients provided written informed consent.

## Author contributions

All authors listed have made a substantial, direct, and intellectual contribution to the work and approved it for publication.

## Funding

This study was supported by the Beijing Medical Award Foundation Project (YXJL-2021-0170-0256), National Natural Science Foundation of China (82072130 and 81873925), 333 High-level Talent Training Project in Jiangsu Province (BRA2020089), and Six Talent Peaks Project in Jiangsu Province (WSN-022). The funders had no role in the study design, data collection, data analysis, interpretation, or writing of the manuscript.

## Conflict of interest

The authors declare that the research was conducted in the absence of any commercial or financial relationships that could be construed as a potential conflict of interest.

## Publisher’s note

All claims expressed in this article are solely those of the authors and do not necessarily represent those of their affiliated organizations, or those of the publisher, the editors and the reviewers. Any product that may be evaluated in this article, or claim that may be made by its manufacturer, is not guaranteed or endorsed by the publisher.

## Footnotes

^1^ www.91trial.com
