# Supplementary material for: Effect of esketamine on postoperative depressive symptoms in patients undergoing thoracoscopic lung cancer surgery: A randomized controlled trial
Source: Front Psychiatry. 2023 Mar 15;14:1128406. doi: 10.3389/fpsyt.2023.1128406 (PMC10050377; doi:10.3389/fpsyt.2023.1128406)
Supplement: Supplementary file 1 [file Table_1.docx]

**Table S1. Details of patients with depressive symptoms at 1 month postoperatively**

|  | **Esketamine** | **Normal saline** | | | | | | | | |
| --- | --- | --- | --- | --- | --- | --- | --- | --- | --- | --- |
|  | Case 1 | Case 1 | Case 2 | Case 3 | Case 4 | Case 5 | Case 6 | Case 7 | Case 8 | Case 9 |
| Preoperatively |  |  |  |  |  |  |  |  |  |  |
| Age | 34 | 58 | 41 | 35 | 61 | 70 | 36 | 70 | 56 | 42 |
| Sex | Female | Female | Male | Female | Female | Female | Female | Female | Female | Female |
| BDI-II scores | 1 | 6 | 9 | 0 | 0 | 0 | 26 | 0 | 0 | 29 |
| Depressive symptoms | No | No | No | No | No | No | Yes | No | No | Yes |
| 1 month postoperatively |  |  |  |  |  |  |  |  |  |  |
| Depression severity | Mild | Mild | Moderate | Mild | Mild | Severe | Mild | Mild | Moderate | Mild |
| BDI-II scores at 1 month |  |  |  |  |  |  |  |  |  |  |
| Total | 14 | 17 | 21 | 18 | 14 | 34 | 16 | 14 | 23 | 16 |
| 1. Sadness | 1 | 1 | 1 | 0 | 1 | 2 | 1 | 1 | 1 | 1 |
| 2. Pessimism | 0 | 1 | 1 | 3 | 1 | 2 | 2 | 0 | 2 | 0 |
| 3. Past failure | 0 | 0 | 3 | 0 | 0 | 1 | 0 | 0 | 0 | 0 |
| 4. Loss of pleasure | 1 | 1 | 1 | 3 | 1 | 3 | 1 | 1 | 1 | 1 |
| 5. Guilty Feelings | 0 | 0 | 2 | 0 | 0 | 2 | 0 | 0 | 0 | 1 |
| 6. Punishment feelings | 0 | 0 | 0 | 0 | 2 | 0 | 0 | 0 | 2 | 1 |
| 7. Self-dislike | 0 | 1 | 1 | 1 | 0 | 2 | 0 | 0 | 1 | 1 |
| 8. Self-criticalness | 0 | 0 | 1 | 0 | 0 | 0 | 0 | 0 | 0 | 0 |
| 9. Suicidal thoughts | 0 | 0 | 0 | 0 | 0 | 0 | 0 | 0 | 0 | 0 |
| 10. Crying | 1 | 1 | 1 | 1 | 0 | 1 | 0 | 1 | 1 | 1 |
| 11. Agitation | 1 | 1 | 1 | 0 | 1 | 1 | 1 | 0 | 1 | 0 |
| 12. Loss of interest | 1 | 0 | 0 | 0 | 1 | 3 | 0 | 0 | 1 | 0 |
| 13. Indecisiveness | 0 | 0 | 0 | 0 | 2 | 1 | 0 | 0 | 1 | 0 |
| 14. Worthlessness | 0 | 1 | 1 | 0 | 0 | 0 | 0 | 0 | 0 | 0 |
| 15. Loss of energy | 3 | 3 | 3 | 3 | 3 | 3 | 3 | 3 | 3 | 3 |
| 16. Changes in sleeping | 1 | 3 | 1 | 2 | 0 | 3 | 3 | 3 | 2 | 3 |
| 17. Irritability | 2 | 0 | 0 | 2 | 0 | 0 | 3 | 0 | 1 | 1 |
| 18. Change in appetite | 0 | 1 | 1 | 1 | 0 | 3 | 0 | 1 | 0 | 1 |
| 19.Concentration difficulty | 1 | 1 | 1 | 1 | 1 | 1 | 1 | 1 | 1 | 1 |
| 20. Tiredness or fatigue | 2 | 2 | 2 | 1 | 1 | 3 | 1 | 3 | 2 | 1 |
| 21. Loss of interest in sex | 0 | 0 | 0 | 0 | 0 | 3 | 0 | 0 | 3 | 0 |

BDI-II, the Beck Depression Inventory-II.

A total BDI-II score of 0–13, 14–19, 20–28, and 29–63 indicates absent, mild, moderate and severe depressive symptoms, respectively.
